# The gender achievement gap in grades and standardised tests—what accounts for gender inequality?

**DOI:** 10.3389/fsoc.2024.1448488

**Published:** 2024-10-16

**Authors:** Hannu Lehti, Markus Laaninen

**Affiliations:** Department of Social Research, Invest Flagship Center, University of Turku, Turku, Finland

**Keywords:** gender, achievement gap, school, grades, test scores

## Abstract

We studied the gender achievement gap in grades and standardised test scores in Finland, where the gender differences are largest among OECD countries. We compared the gender achievement gap in standardised test scores from PISA surveys and grades from high-quality school registers in literacy. Furthermore, we analysed how grades differ from standardised test scores by family background and students’ SES composition of the schools. By using the Blinder-Oaxaca decomposition method, we explored how different characteristics between girls and boys explain gender differences in grading. Our findings indicate that boys’ grades were lower than can be expected based on standardised test scores. The gender gap in grades was explained by boys’ lower reading interests, effort put into schoolwork, and conscientiousness on homework. However, even adjusting for schooling characteristics and competence, boys have lower grades than test scores in schools that have low SES student composition.

## 1 Introduction

Girls’ school performance exceeds that of boys in all Western countries (Voyer and Voyer, 2014). The gender achievement gap in learning outcomes has been attributed to boys’ lower non-cognitive traits and social and behavioural skills (Buchmann and DiPrete, 2006; Buchmann et al., 2008). On average, girls invest more in schooling, they are more conscientious, self-disciplined, motivated to go to school, and their educational aspirations and effort put into schoolwork are higher than boys (Di Prete and Jennings, 2012; Duckworth and Seligman, 2006; Entwisle et al., 2007; Heyder and Kessels, 2017; Lam et al., 2012; Trautwein et al., 2006). Boys, on average, lack intrinsic motivation, are more often diagnosed with learning difficulties, are more likely to feel alienated at school, and boys’ behaviour in school is more sanctioned (Hascher and Hagenauer, 2010; Trzesniewski et al., 2006).

In addition to individual-level non-cognitive traits, fewer attention has been given to social structures within the school that may also explain part of the gender achievement gap (e.g., Downey and Vogt Yuan, 2005). The norms that are valued in schools, such as obedience, conscientiousness, and aspiration for learning, are more typical among girls than boys (Autor et al., 2019).

Previous studies have shown that the gender achievement gap is wider in teacher evaluations (school grades) than in non-teachers evaluated standardised tests favouring girls over boys (e.g., Protivínský and Münich, 2018; Lievore and Triventi, 2023; Bonesrønning, 2008; Cornwell et al., 2013; Falch and Naper, 2013; Lindahl, 2007). Studies indicate that boys are more sensitive to learning circumstances than girls, influencing boys’ learning outcomes more than girls’; therefore, the learning gap tends to be wider in low SES families compared to high SES families (Autor et al., 2019; Stienstra and Karlson, 2023; Salminen and Lehti, 2023). However, studies have not thoroughly investigated the effects of schools’ student composition on gender achievement gaps in grades and standardised test scores.

The focus and contribution of this article are to analyse how gender and schools’ SES composition intersect in the learning outcomes. Although previous studies have analysed the gender achievement gap, it has not considered how contextual factors are associated with the gap. In this article, we expand the focus by analysing different mechanisms that may explain the gender achievement gap in grading and how the gender achievement gap interacts with schools’ SES composition. More specifically, first, we examine whether the gender achievement gap is wider in grades than in standardised test scores, as previous studies have suggested (Protivínský and Münich, 2018). Second, we analyse how much non-cognitive skills, i.e., effort put into schoolwork, and motivational factors explain the gender gap in grades. Third, we study whether SES composition in schools is a more important explanatory factor than family SES for gender gap in grades.

We use Programme for International Student Assessment (PISA) and high-quality full population school register data from Finland to compare non-teacher-evaluated PISA scores in literacy with teacher-evaluated literacy within the same study cohort. By comparing these datasets, we study whether boys’ and girls’ grades predict their PISA achievements.

## 2 Theoretical background

### 2.1 Gender grading gap and grading bias

The gender achievement gap means the difference between girls’ and boys’ learning outcomes. Girls have an advantage in a range of indicators of academic performance over boys, particularly in literacy (Voyer and Voyer, 2014; Autor et al., 2019). Girls outperform boys in PISA literacy tests in all participating countries in every test year. Girls are also more interested in reading and more motivated to read books than boys (Houtte, 2004).

Gendered grading bias means differences in grades between girl and boy students with the same level of academic skills (e.g., Protivínský and Münich, 2018). Several studies have shown that boys get lower grades and test scores from their teachers than in non-teacher-evaluated tests. Biases in grading against boys were found in literacy and math in studies that compared teacher-given grades to anonymously evaluated test scores in both US and European contexts (Bonesrønning, 2008; Cornwell et al., 2013; Falch and Naper, 2013; Lindahl, 2007; Protivínský and Münich, 2018). For example, in a study conducted in Israel, boys faced grade discrimination in literacy, math, and science while comparing blind and non-blind evaluated primary school test scores (Lavy, 2008). The literacy review by Protivínský and Münich (2018) showed that only 2 of 13 studies did not find gendered grading bias against boys; however, the methodology of these two studies was different because teachers evaluated exams with a particular gender, age, and caste assigned (see Hanna and Linden, 2012).

It has been suggested that grading bias emerges from the teachers’ stereotypical perception of genders (Lavy, 2008) but also from socioeconomic background (Speybroeck et al., 2012). Teachers may use stereotypes of certain groups of students as shortcuts to process information more easily and efficiently (Bordalo et al., 2016).

The above-mentioned empirical evidence shows that the gender achievement gap in teachers’ evaluated grades may be wider than in standardised test scores that teachers do not evaluate. Thus, we assume that *the gender achievement gap is smaller in PISA tests when compared to teacher-evaluated grades in literacy* (H1). This means that boys score better in standardised tests than teacher-evaluated grades, and girls’ grades are better than their standardised test scores.

### 2.2 Non-cognitive traits, grading, and standardised test scores

If female-typical behaviour is valued in school, it may lead to a bias against boys, as school grades are influenced by non-cognitive factors like behaviour (Cornwell et al., 2013; Di Prete and Jennings, 2012; Robinson-Cimpian et al., 2014).

Teachers may account for academic effort and motivation in their grading, although the competence and performance of boy and girl students may be the same (Di Prete and Jennings, 2012; McMillan, 2001; Randall and Engelhard, 2010). Moreover, teachers tend to perceive girls as more motivated (e.g., Gentrup and Rjosk, 2018), put more effort (Downey and Vogt Yuan, 2005), are better behaving (Glock and Kleen, 2017), and produce less disruptive behaviour (Downey and Vogt Yuan, 2005; Terrier, 2020). However, Matějů and Smith (2015) found that girls outperform boys in literature grades, but the whole gap is not explained by ability in reading (PISA test scores) and attributes such as self-efficacy or troubled behaviour. According to Duckworth and Seligman (2006), non-cognitive traits that are on average more female-typical than male, such as self-discipline, did explain the gender grading gap, but these non-cognitive skills did not have an influence on standardised test scores or IQ tests between boys and girls. A similar pattern was observed by Cornwell et al. (2013). According to these studies, it seems that non-cognitive traits have less influence on the gender gap in standardised test scores than school grades (Protivínský and Münich, 2018). However, previous research has also shown that boys are overrepresented in populations with reading disabilities, antisocial behaviour, mental retardation, attention disorders, dyslexia, and delayed speech that influence not only their grading but test scores (Buchmann et al., 2008). Girls typically exhibit earlier social and biological development compared to boys, leading the advantages in orientation to learning during the elementary school years in early childhood and youth. Some studies have found that girls spend more time than boys in outside-of-school activities that promote reading skills and therefore higher standardised test scores in reading, whereas boys participate in outside-of-school activities that promote math skills (Downey and Vogt Yuan, 2005).

Experimental studies show that students who display higher effort tend to influence teachers’ perception of their higher achievement, although effort and achievement may not be associated with each other (Brookhart, 1993; Randall and Engelhard, 2010). Ethnographic studies have explained the gender gap in an effort by different school cultures of boys and girls. In the boys’ peer culture, it appears to do little or no work for school, whereas in the girls’ peer culture, working hard at school is not only accepted but also desirable (Epstein, 1998; Morris, 2008). Because boys tend to show effortlessness, investing less in schoolwork on average than girls, teachers may see such effortlessness as typical among boys but not so much among girls (Heyder and Kessels, 2017).

This implies that all else being equal while taking the effort into account, the gender gap in teachers’ grading should be reduced. If girls’ higher effort and motivation would be associated with higher grades, then when considering academic motivation and effort, the gender achievement gap in teacher-given grades would reduce or diminish entirely. However, the effort would not influence standardised test results (that teachers do not evaluate) of equally competent students. Therefore, we study whether the motivation for reading and effort put into school work explain the gender achievement gap in literacy grades. We assume that *the gender achievement gap in teacher-given grades would reduce when we control for the effort put into schoolwork, school competence and motivational factors* (H2).

### 2.3 Family background and gender achievement gap

The theory of subculture (Cohen, 1955) states that school alienation is a reaction against school norms among working-class boys’ whose needs are not fulfilled at school. It is an expression of resistance to school. In his seminal ethnographic work, Willis (1978) emphasised that working-class boys do not value school-valued feminine traits like diligence and good behaviour. Because working-class boys do not identify with the culture and norms of the school, they may form anti-school culture. Alienation from school norms explains why working-class boys’ achievement in school is lower than middle-class students. Ethnographic studies have found support for the theory that particularly lower SES boys’ conceptions of masculinity conflict with academic achievement because they cannot use academic achievement to succeed in the working-class occupations that they are accustomed to in their family sphere (Morris, 2008; Epstein, 1998).

If school alienation theory applies only to low SES working-class boys, the gender gap in school achievement would be large in low SES families. However, it would be narrow or disappear between girls and boys from high SES families. High socioeconomic family background may be a protective factor for boys, which may result in a smaller gender achievement gap among students from high SES families. In the USA, for example, a study found that boys’ reading skills were lower than girls only among children from low SES families (Entwisle et al., 2007). Based on theories on low SES boys’ anti-school characteristics, we assume that *the gender achievement gap in standardised test scores and school grades is greater among low SES than high SES children* (H3a).

The interaction between gender and family SES on learning outcomes can also be examined from the perspective of internalised anticipated values and virtues that schools expect from the students (Bourdieu and Passeron, 1990). The cultural habits of the middle class are evident in the values and norms that schools appreciate. Studies indicate that teachers’ grading is influenced by the students’ cultural practices. Jæger and Møllegaard (2017) showed that teachers evaluated grades higher for identical twins with higher cultural capital, although no differences were detected in twins’ non-teacher-evaluated grades.

However, these previous studies have not considered how gender and family SES together influence achievement. The emphasis on non-cognitive traits by teachers can be particularly pronounced among boys from low SES (working-class) backgrounds, meaning that grading of the low SES boys does not reflect their actual competencies but more likely their non-conformity in schools. Low SES girls may compensate for their lower background in schools through their girl-typical characteristics such as conscientiousness and self-discipline (Buchmann and DiPrete, 2006; Buchmann et al., 2008).

According to theory, it can be assumed that boy (and girl) students with high SES backgrounds have internalised normative behaviour that the school expects; their grading is not similarly affected by non-conformity as low SES boys. The teachers’ grading bias can be found between girls and boys from low SES families but not among those from high SES ones. Therefore, *the moderation effects of the family background on the gender achievement gap are greater in grades when compared to standardised test scores* (H3b).

### 2.4 Schools’ SES composition of the students

In addition to the SES background, the SES composition of students in schools can shape anti-school attitudes and the constructed masculinity of boys’ peer cultures and thus influence boys’ orientation towards school. It has been shown that in a school context with a high proportion of students from a low socioeconomic background, disciplinary problems are more frequently present (Lievore and Triventi, 2023; Rindermann, 2007). On the other hand, high SES students’ composition in school can create a learning-oriented environment, which increases the appreciation of academic achievement among adolescent males and facilitates commitment to schoolwork. Girls’ peer groups, by contrast, do not vary as strongly with the social environment in the extent to which they encourage academic engagement, and girls are less likely to be stigmatised for school engagement (Legewie and DiPrete, 2012).

The school’s social environment may influence the learning outcomes of boys more than girls. Boys gain more than girls from a high SES learning-oriented environment because it channels how masculinity is constructed in the school culture. Such an environment can promote academic competition as an aspect of masculinity because academic learning is more valued among high SES students than low SES students. Although it has been recognised that school context can influence students’ learning outcomes, there are only a few quantitative studies that have analysed school context. Legewie and DiPrete (2012) found that the SES composition of the schools indeed influenced boys’ learning outcomes more than girls, even after controlling for parental SES. The gender achievement gap was larger in schools that had more low SES students than high SES students. Lievore and Triventi (2023) found that teacher and classroom characteristics such as the percentage of female students, students’ SES composition, or classroom size did not reduce grading premium favouring girls over boys.

Not only boys’ masculine anti-school attitudes and behaviour may explain the effects of SES composition, as previous literature has theorised. One explanation is that the composition of students’ SES can influence teachers’ grading because the teachers’ bias can be stronger among schools that have a higher proportion of lower than higher SES students. Thus, stereotypes related to gender and low SES background may influence teachers’ grading more in schools with a higher proportion of low SES students. Teachers may give lower grades for students showing boy-typical behaviour in schools with low SES students. This may emerge because boy students in lower SES schools compared to high SES schools display more unsuitable school behaviour that teachers associate with the low cultural capital of the working-class background (see, e.g., Willis, 1978). Although not all the boys have low SES cultural habits—or not even low SES background—in schools with a high proportion of low SES students, teachers’ stereotypical beliefs can have a stronger influence on the grading of boy students. Boys may have lower grades in low SES schools because their habits and behavioural patterns are stereotypically believed by the teachers to be boy-typical, although boys’ characteristics in competence, motivation, and effort would be the same as girls. Therefore, we expect that *the difference in boys’ and girls’ grades is smaller in high SES schools compared to low SES schools, and the gender gap in standardised test scores does not differ according to students’ SES composition of the schools* (H4).

If schools’ SES composition would have the same influence on the gender gap in standardised test scores and teachers’ given grades, then masculine anti-school attitudes in the low SES schools would explain the effect on boys’ learning outcomes.

## 3 Materials and methods

We computed analyses using register and PISA data from Finland. In the Appendix, we have described the country context of the study under the title: The country context of the study.

### 3.1 PISA data

We used PISA data from 2000, 2003, 2006, 2009, 2012, and 2015. The target population of the Finnish PISA survey comprised students born between February and January, who were approximately 15 years of age when the survey was conducted. In the random sampling process, schools were selected first, followed by the students. The sample considered the regional and size-based representativeness of the schools (Nissinen et al., 2018).

We excluded students who were born in January (1 year younger) from the PISA data (4–8% yearly) that PISA and register data corresponded exactly to the same cohorts. The analytical sample size for PISA was 26,702 students.

In the background survey of the PISA, students are asked about their parents’ profession. The students write about their parents’ main job and describe what they do as part of their job. Based on these responses, the parents’ occupations are defined.

As we used pooled PISA data, the years with greater sample sizes got more weight in the analyses (see Appendix Table 1A). However, the gender achievement gap stayed relatively stable in both PISA and register data over the years, so different sample sizes did not affect our results (Appendix Figure 1A).

### 3.2 Register data

The register data are based on the Finnish personal register data, which comprises all data on people living in Finland from 1987 to 2016. We also used secondary school joint application registers, from which we obtained information on the grades as recorded in the compulsory school leaving certificates of the birth cohorts who participated in the PISA surveys (born 1984, 1987, 1990, 1993, 1996, and 1999).

Data on the parents’ occupations were taken from the employment register data. Occupational register data are available for 1990, 1993, 1995, 2000, and 2004–2016; thus, it contained missing data for certain years. Parents may also be unemployed or out of the labour market. As a study utilising Finnish register data has shown that the occupations of parents do not change significantly after the birth of a child (Erola et al., 2016), we took the occupation of a parent for the first possible year when it was available when the child was aged under 17 years. Data on the parents’ occupations were available for about 90% of the children when they were aged 10–16 years.

Each year, about 4–5% of the students had missing data in the registers. The register lacked information on grades if the student did not apply to secondary education or applied to a secondary educational institution that was outside Finland. No differences were detected between the missing values of girl and boy students. In total, over all the birth cohorts, we observed that on average, 10.8% of girls and 10.23% of boys have not had grades marked in the registers. We excluded the year 2009 from the analysis because secondary school joint register data were not completed at that year. The analytical sample of register data included 283,677 students.

### 3.3 Variables

In the register data, our dependent variable is the grades given by the teacher for literacy, as derived from the compulsory school leaving certificate, which is awarded when students are 15 years of age. This was the same school year they participated in the PISA tests. The grades in Finland range from 4 (rejected) to 10 (excellent). In the PISA data, we used plausible value (PV) scores that we derived from the test results in literacy (OECD, 2017) as our dependent variable. Furthermore, when we analysed mechanisms with Blinder-Oaxaca modelling, we used the literacy grade reported by the students as our dependent variable in PISA 2000 data. We *z*-standardised all the dependent variables so that test results and grades are comparable. The mean of the total samples was 0 and the standard deviation was 1.

**Figure 1:**
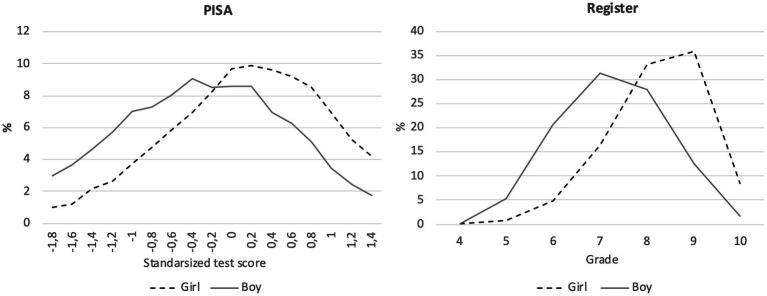
Literacy distributions by gender in PISA (test scores) and school register data (grades).

In Figure 1, we show the distribution of the grades and PISA test results. Table 1A shows the means and standard deviations of the applied variables in the analyses. Figure 1 shows that girls fared much better in literacy according to both PISA and register datasets.

We measured the parental SES with the ISEI index, which we derived from the ISCO classification; its values ranged from 16 to 90 points (Ganzeboom et al., 1992). ISEI scores form a scale of occupations which is constructed by regressing individuals’ occupations with income and education, making it closely related to all the widely used socioeconomic status indicators. Other studies that analysed the gender learning gap by family SES have used the same indicator to measure family SES (Legewie and DiPrete, 2012). We used the dominance principle meaning that the value of the ISEI variable corresponded to the information on the parent (mother or father) with a higher ISEI. We *z*-standardised and classified parental ISEI into deciles by year in the register and PISA data to match the distribution in the register and weighted PISA data. We did not use parental education as Finland’s PISA surveys include severe measurement errors owing to reporting bias among the students (Lehti and Laaninen, 2021).

We used as control variables students’ birth cohorts (dummy for each year) and students’ immigration status (dummy) as the gender gap may vary between students with different cultural backgrounds (Nollenberger et al., 2016). The parental ISEI is controlled for in the main models because there is some evidence that parental SES may have different influences on the achievements of boys and girls (Hopcroft and Martin, 2016).

In decomposition analysis, we study whether variation in student effort, motivation in reading, and composition of students’ SES in schools explain the gendered grading gap. In this part, we use the PISA 2000 sample because it includes both students’ literary grades and PISA test scores. We measured a student’s effort with two variables that were found in the PISA 2000: whether students conducted their homework on time (HOMEWORK) and the index of effort and perseverance (EFFPER). The HOMEWORK variable was a categorical variable grouped as never (1), sometimes (2), most of the time (3), and always (4). The index of effort and perseverance (EFFPER) is a composite measure of four variables to measure how much effort students put into schoolwork. Because it can be assumed that one of the explanatory factors between boys’ and girls’ grades can be motivation for reading, we analyse how much interest in reading (INTREA) explains the grading gap between boys and girls. The variable is a composite measure of four variables. INTREA as well as EFFPER was created by using principal component analysis and is a *z*-standardised continuous variable. Finally, we analyse whether the composition of the students’ SES in the schools matters for teachers’ grading by taking the average of the parental ISEI according to schools (Legewie and Diprete, 2012). Because in Blinder-Oaxaca models each variable must have a zero point, we reduced the minimum value (ISEI = 16) from the parental ISEI and schools’ average parental ISEI variables (Jann, 2008). All the variables are described in a more detailed manner in Appendix Table A3. In Appendix Table 7A, we show the correlation matrix between all the variables that were used in the analyses. It shows that there are no high collinearities between used variables.

### 3.4 Methods

To calculate the gender achievement gap in PISA and register, we used linear regression models where we controlled for parental ISEI in quadratic form, students’ immigration status, and the birth cohort. However, we did not find significant differences by birth cohorts (see Appendix Figure 1A). In the analysis of PISA data, we utilised the Repest package written for the Stata programme. Repest considers the hierarchy and sampling of the data and the use of PV values in the analysis (Avvisati and Keslair, 2014). Therefore, the hierarchical structure of the data, students nested into schools (school fixed effect), is accounted for when standard errors are calculated.

The results can be interpreted as the average difference-in-difference estimate (difference between boys and girls across registers and PISA results) on students’ cohort level and show how much larger the gender gap is in school grades when compared to PISA. If there is a negative estimate (difference between girls and boys), it shows that the gendered grading gap is larger than the gender gap in PISA results.

The analysis part is arranged in the following way: *first*, in the descriptive part, we study gender achievement gaps in PISA test scores and teachers’ evaluated grades and compare gender achievement gaps between PISA tests and grades across the years and by family background.

Then, we analyse the mechanisms behind the gender achievement gap differences in grading and standardised scores. We cannot use the register dataset in this part because it did not include variables on students’ effort or motivation.^1^ Because the PISA 2000 questionnaire includes students’ literacy grades, we conducted detailed 2-fold Blinder-Oaxaca models and linear regression interaction models by using only the PISA 2000 sample. We analysed whether effort, motivation for reading, and students’ school composition explained average literacy grades between girls and boys (the gender achievement gap in grades). The models that we used in the decomposition part are described in Equation 1:


(1) $$\begin{array}{l} \Delta \bar{Y}=\overset{k}{\underset{j=1}{\sum }}\beta_{j}^{2}\left({\bar{x}}_{j}^{1}-{\bar{x}}_{j}^{2}\right)+\overset{k}{\underset{j=1}{\sum }}{\bar{x}}_{j}^{2}\left(\beta_{j}^{1}-\beta_{j}^{2}\right)\\ D\;=\;E\;+\;U \end{array}$$

This is a 2-fold decomposition of the difference in means predicted outcome. The explained component E indicates the change in the group mean predicted outcome when it meets the reference group covariates level. In our case, it shows how much boys’ literacy grades change when the effort put into schoolwork is on the same level (mean) as girls’ effort put on schoolwork. The gap explained is usually referred to as the endowment effect because it shows the effect on outcome when boys’ “endowments”—for example, the effort put on schoolwork—are the same level as for girls. If there are no differences in the mean of explanatory variables, i.e., endowments between girls and boys, explained component E cannot explain the predicted outcome.

The unexplained component U shows the differential effect of observable variables and the level of unobservable variables. The variables that have different regression coefficients ( β ) on girls’ and boys’ grades are displayed in the U component. The U component can be interpreted as a change in girls’ mean grades when girls have the same regression coefficients as boys. In addition, it shows an unexplained gender achievement gap in grades between girls and boys.

The 2-fold decomposition was used instead of the 3-fold models because there was no significant interaction effect (U and E components).

## 4 Results

### 4.1 The gender achievement gap in test scores and grades across the years

**Figure 2:**
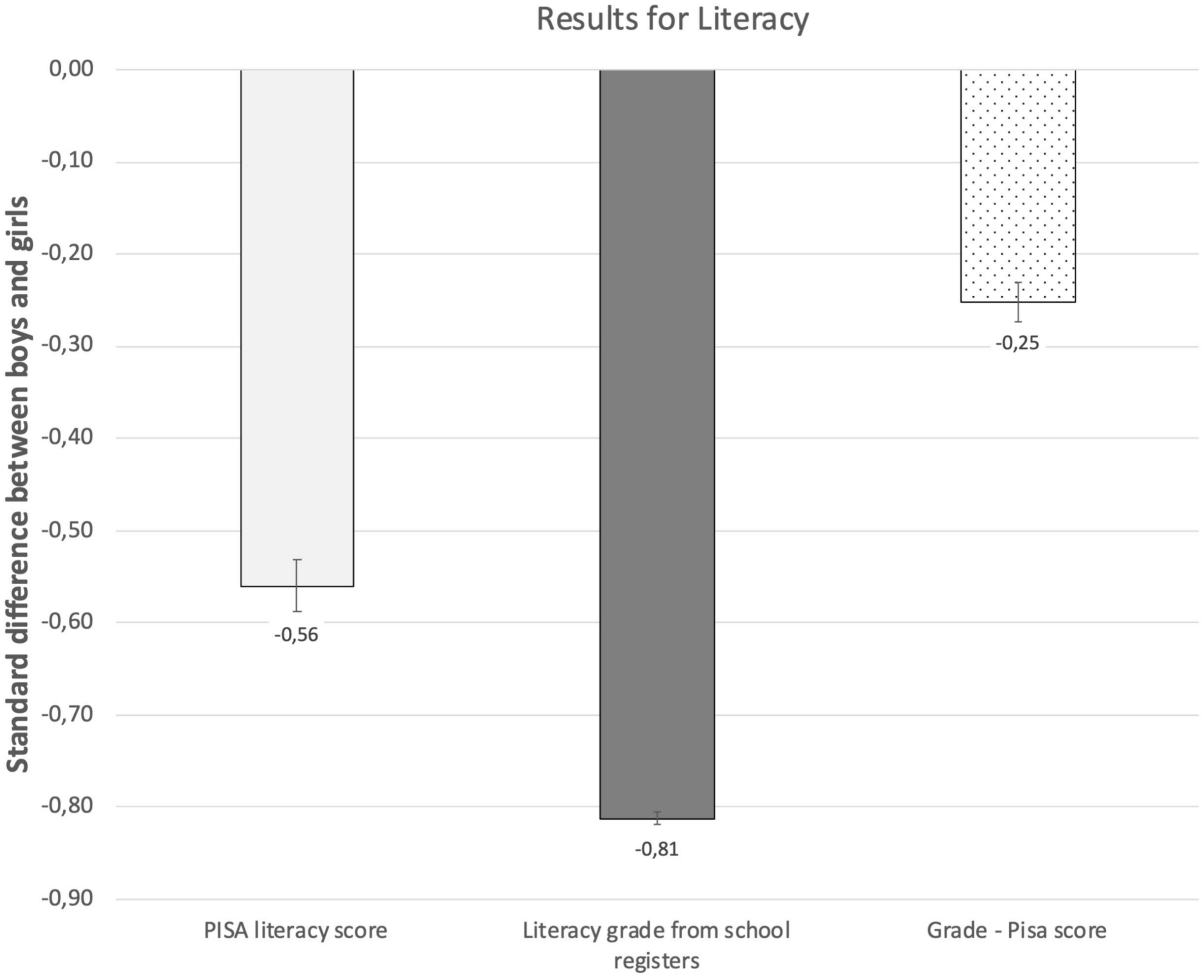
The gender achievement gap (boys’ values reduced from the girls’ values) in PISA test scores and school grades in literacy. Estimates are *z*-standardised and 95% CIs are around the estimates.

Figure 2 shows that girls have higher achievement than boys in literacy. In the PISA literacy scores the difference for girls was 0.56 SD and in the grades the difference was 0.81 SD. The results are in line with our hypothesis that the gender achievement gap is smaller in standardised tests than in teachers’ given grades (H1). PISA scores and school grades thus show very different pictures of the gender achievement gap. The difference in the gender achievement gap between the PISA scores and grades was 0.25 SD (see the numbers in Appendix Table 2A). The gender achievement gap remained stable over the years in both PISA and register data (Appendix Figure 1A).

### 4.2 Which students’ characteristics explain the gender achievement gap?

Next, we analyse the gender achievement gap in more detailed matters. In this part, we test hypothesis 2 that the gender achievement gap in grades would reduce when we control for the effort put into schoolwork, school competence, and motivational factors. Table 1 shows the results of detailed Blinder-Oaxaca decomposition models. In model 1, we analysed whether schools’ SES composition explains the gender gap in grading when parental SES is also controlled for. Because the mean students’ SES composition of the schools is the same for girls and boys (see Appendix Table 3A), there cannot be a difference in endowment effect. However, the coefficient is −0.12 and statistically significant. This means that boys reach girls’ literacy grades when schools’ SES composition increases. The coefficient for parental SES is not statistically significant in model 1, although the coefficient seems to be the same as students’ SES composition.

**Table 1:** The detailed decomposition results of the Blinder-Oaxaca models analyse the difference between boys’ and girls’ literacy grades.

	Model 1		Model 2		Model 3	
Variables	Endowment	Coefficient	Endowment	Coefficient	Endowment	Coefficient
Parental ISEI school mean	0.00	−0.12*	0.00	−0.06	0.00	−0.06
	*0*.*00*	*0*.*06*	*0*.*00*	*0*.*05*	*0*.*00*	*0*.*05*
Parental ISEI	−0.01	−0.12	0.00	−0.07	0.00	−0.08
	*0*.*01*	*0*.*07*	*0*.*00*	*0*.*06*	*0*.*00*	*0*.*06*
INTREA			0.16***	−0.01	0.07***	−0.01
			*0*.*01*	*0*.*00*	*0*.*02*	*0*.*01*
EFFPER			0.05***	0.00	0.04***	0.00
			*0*.*01*	*0*.*00*	*0*.*01*	*0*.*00*
HOMEWORK			0.05***	0.02	0.05***	0.07
			*0*.*01*	*0*.*10*	*0*.*01*	*0*.*09*
PISA score literacy (std)					0.19***	0.00
					*0*.*03*	*0*.*00*
	Explained	Unexplained	Explained	Unexplained	Explained	Unexplained
Difference (girls vs. boys)	−0.01	0.78***	0.27***	0.52***	0.34***	0.43***
	*0*.*01*	*0*.*03*	*0*.*02*	*0*.*03*	*0*.*03*	*0*.*03*
Total %	0	100.8	32.9	67.1	43.8	55.3
Total difference (girls vs. boys)	0.777***					
	0.029					

All models control for immigration background. *N* = 4,216, standard errors in italics. *p < 0.05, **p < 0.01, ***p < 0.001.

In the next model, when we also control for homework, effort, and interest in reading, the school’s SES composition is not statistically significant anymore, and the coefficient is reduced by half to −0.06. This means that in schools where students’ average SES is higher, the students are more motivated to read, do homework on time, and show more effort in schoolwork than in the schools where students’ average SES is low. Furthermore, model 2 shows that if boys’ reading interest (INTREA), effort (EFFPER), and conscientiousness in homework (HOMEWORK) were the same as girls’, the gap between girls’ and boys’ grades would be reduced by 0.27, being 0.52 standard deviations. Boys’ lower interest in reading explains most of the results. If boys’ reading interests were at the same level as girls, it would increase their literacy grades by 0.16 standard deviations. There is no coefficient association in these variables, which means these variables increase literacy grades for both genders.

The gender achievement gap in PISA scores was measured to be 0.25 SD smaller than the grading gap (see Figure 1). However, in model 2 when considering variables that measure homework, effort, and interest in reading, this achievement gap grading bias for the boys disappears. This means that if these factors were at the same level for boys as girls, the grading gap as well as the gap found in the PISA results would be the same, and there would not be any grading bias.

Finally, in model 3, we also consider the PISA reading score, which explains about half of the endowment effect of reading interest (those who are more interested in reading have higher PISA scores), but controlling for the PISA score does not explain away the endowment effect for effort or homework conducted in time. These results indicate that girls get higher grades compared to PISA test results because they show more effort in schoolwork, they are more interested in reading activities, and they are more conscientious about doing homework. Although we control for PISA test results, still these factors influence the grade gap between girls and boys. In model 3, it can also be observed that the coefficient effect is the same for girls and boys, meaning that the regression coefficient of the PISA score increases the same amount of the literacy grades among girls and boys.

Even considering competence (PISA score), it does not explain away the gender gap in grading entirely because still, 55% of the gender gap in literacy grades remains unexplained.

### 4.3 The gender achievement gap by family background

**Figure 3:**
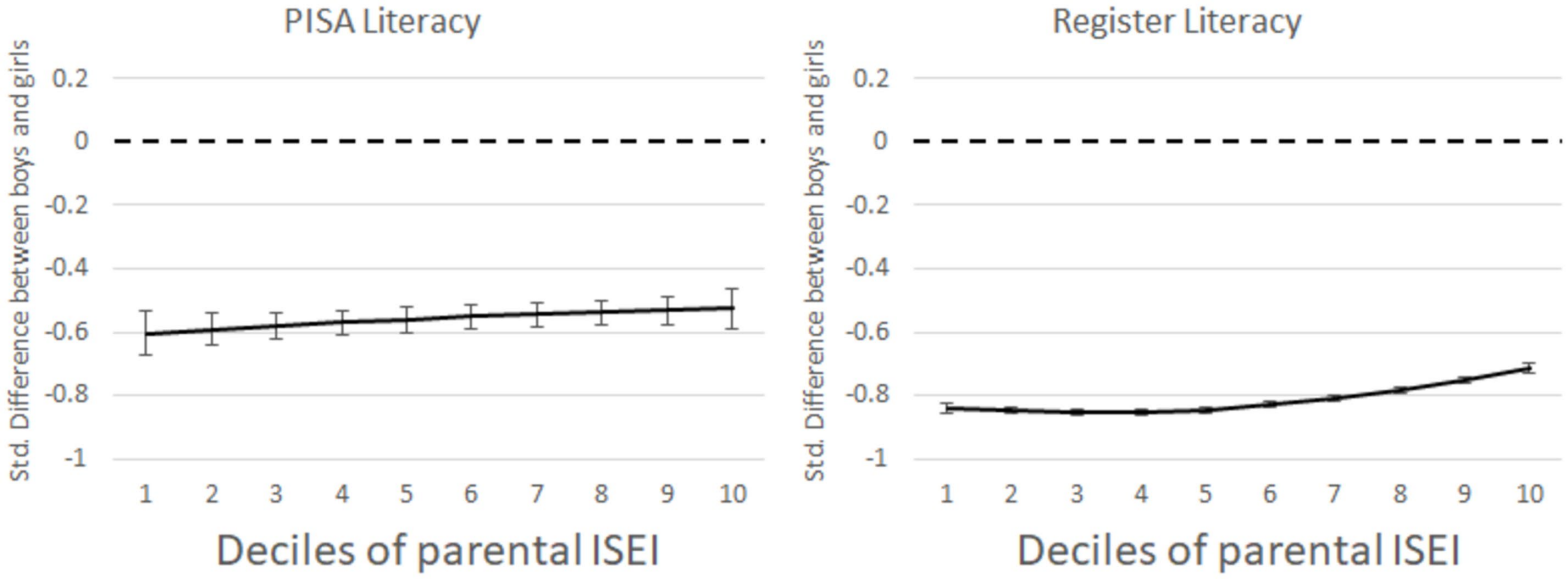
Gender differences in PISA results and teachers’ given grades in literacy by family background (Deciles of parental ISEI). Estimates are *z*-standardised and 95% CIs are around the estimates.

Hypothesis 3a stated that the gender achievement gap would be greater among low SES children when compared to high SES ones. Figure 3 shows the gender gap in PISA scores and grades by family background. Although family SES is positively associated with achievement, it does not show that the gender gap between girls and boys changes much along with the distribution of students’ backgrounds. According to the PISA results, the gender achievement gap at the upper end of parental status was 0.08 SD lower than that at the lower end. In the register, the difference was approximately 0.13 SD. The difference along the parental SES distribution can be considered only marginal.

Because the moderation was stronger in the grades than in test scores but the difference in difference is statistically barely significant but substantially marginal (see Appendix 3A), we concluded that hypothesis 3b cannot be fully supported by the data.

### 4.4 Moderation by school composition

Hypothesis 4 stated that the difference in boys’ and girls’ grades is smaller in high SES than in low SES schools, and the gender gap in standardised test scores does not differ according to students’ SES composition of the schools. Model controls for interest in reading, effort, homework conducted on time, and students’ parental ISEI and PISA test scores. The model shows whether the teachers’ grading depends on the SES composition of the school for boys, although we control for the endowments and competence (PISA test results) that differ between boys and girls.

**Figure 4:**
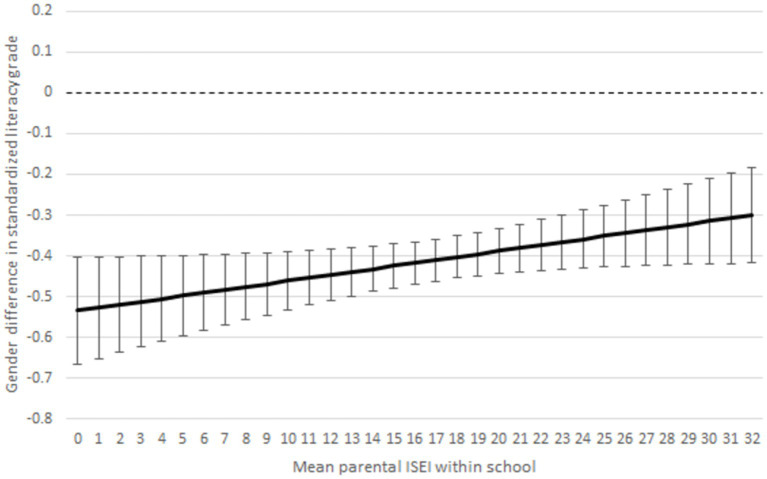
Gender difference in literacy by schools’ student SES composition (mean parental ISEI within school). Note estimates are *z*-standardised and 95% of CIs are around the estimates. Interaction model controls for parental ISEI, immigrant background, index of interest in reading (INTREA), index of effort and perseverance (EFFPER), doing homework on time (HOMEWORK), and PISA test score.

Figure 4 displays that boys, but not girls, have higher grades when students’ SES composition is higher. The result is statistically significant (see Appendix Table 4A). However, girls’ grades are unchanged. Girls’ and boys’ grades converge, although we controlled for PISA test scores and other observed measured factors. Because we cannot explain this result away by using our independent variables, there must be some unobserved variable that explains the results. One unobserved factor is low SES boys’ anti-school behaviour; however, it can be assumed that anti-school masculine behaviour correlates largely with the factors we controlled for in the model, particularly because we controlled for PISA test results and effort put into schoolwork.

**Figure 5:**
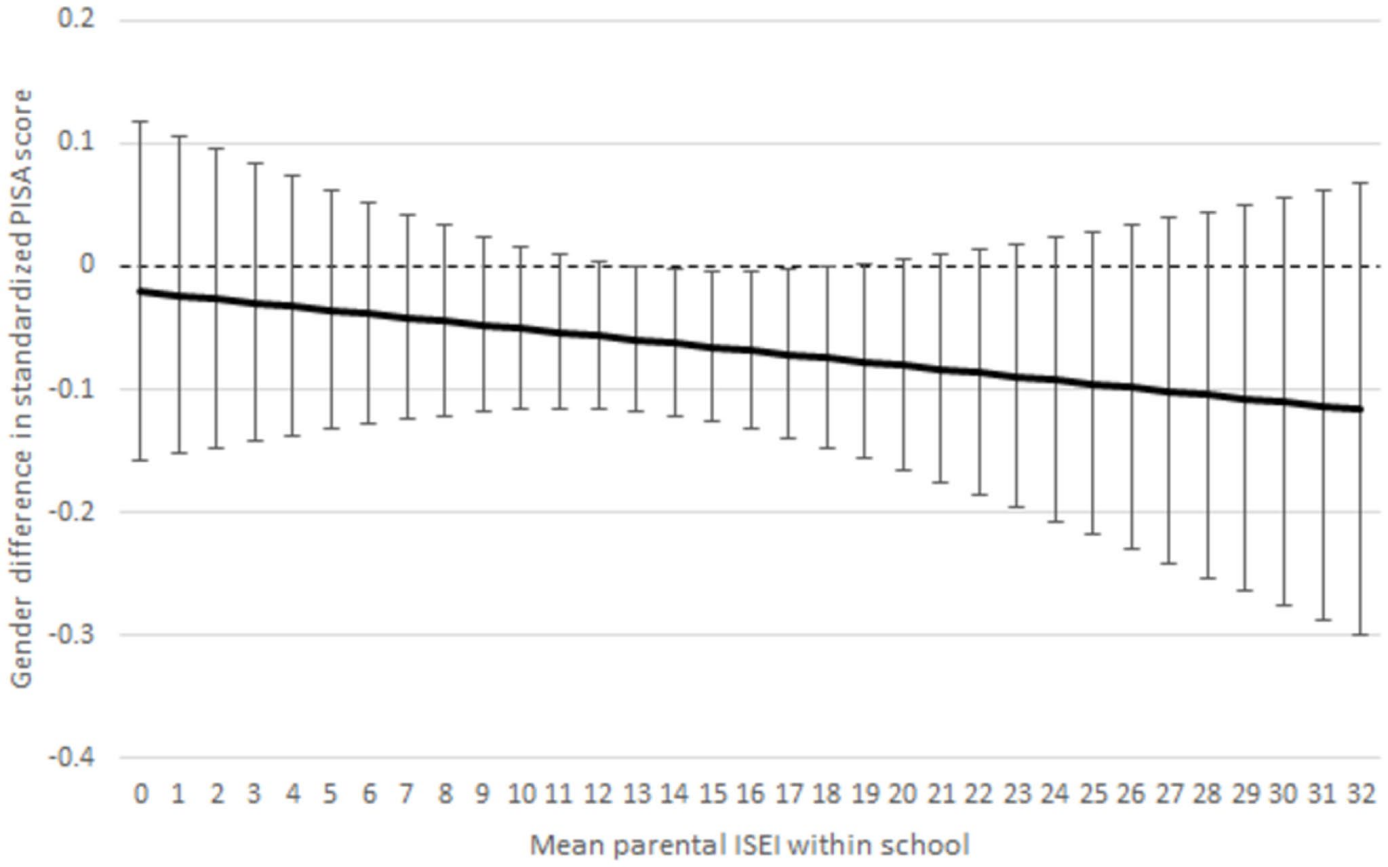
Gender difference in standardised PISA literacy test scores by schools’ student SES composition (mean parental ISEI within school). Estimates are *z*-standardised and 95% CIs are around the estimates. Interaction model controls for parental ISEI, immigrant background, index of interest in reading (INTREA), index of effort and perseverance (EFFPER), and doing homework on time (HOMEWORK).

Furthermore, we conducted a similar linear regression interaction model, but our dependent variable was PISA literacy test scores (Figure 5 and Appendix Table 5A). We did this because it can be assumed that boys get lower *grades* and *test scores* in schools where student SES composition is low because of masculine anti-school attitudes. However, Figure 5 (Appendix Table 5A) does not support the assumption that boys’ (or girls’) PISA literacy test scores would be affected by the SES composition of the schools because the interaction effects are statistically insignificant. The results support hypothesis 4 that the SES composition of the schools modifies only the gender achievement gap in grades but not standardised test scores. In Appendix Table 6A, we compare PISA literacy scores and literacy grades of girls and boys by school SES. The table shows a clear pattern that in schools with higher SES composition, the gendered grading gap is smaller (Appendix Table 6A).

### 4.5 Robustness tests of the results

As the PISA 2000 questionnaire included a question on students’ literacy grades, we conducted a robustness check comparing the PISA 2000 sample to the school register. Appendix Figure 2A shows that there were no significant differences in the gender achievement gap between student-reported or register-based grades.

It may be that boys performed relatively better than girls in the PISA test environment, which is why we see different achievement gaps in PISA tests and school grades. Thus, the difference would not emerge because boys underachieve, and girls overachieve grades considering their competence in PISA tests. The gender achievement gap may have emerged from the difference in the assessments compared. However, Appendix Figure 4A shows that girls and boys got similar grades with similar PISA test success. The differences between boys’ and girls’ PISA scores were statistically insignificant (*p* > 0.05) across all grades.

One mechanism that can play a role is stereotype threat. A stereotype threat is a situation where a group of people is treated negatively because negative assumptions are made about them (Spencer et al., 2016). For example, boys or girls get lower scores on a test that they think is gender-atypical for them. Boys may have a stereotype threat in literacy, whereas girls might have a stereotype boost in literacy. To test this, we also studied the gender gap in math grades and math PISA test scores. We argue based on previous research that girls would have a higher probability of stereotype threat in math, and therefore they would get lower test scores. On the contrary, boys would have a higher probability of stereotype threat in literacy.

However, for maths, we found very similar gender achievement gaps as for literacy. Our findings indicate that (see Appendix Table 2A), although girls’ PISA results in maths are somewhat lower (−0.0215–0.0988 = −0.12) than their grades would predict and higher for boys (0.0798−(−0.0853) = 0.16), the same can also be observed for between grades and literacy PISA test scores [girls: 0.325–0.422 = −0.097, boys: −0.235−(−0.39) = 0.155]. However, in the literacy, PISA tests girls barely have stereotype threat, but boys may have. If stereotype threat were to explain the results, the logical outcome would be that boys’ PISA literacy scores would be lower compared to the literacy grades. However, the results point in the opposite direction. Thus, stereotype threat is hardly the explanation behind the different gender gaps between standardised tests and grades.

## 5 Discussion

We studied how the gender gap in achievement differed between standardised PISA tests and grades assessed by teachers. We explored how different characteristics between the girls and boys explain gender differences in grading and analysed how teachers’ given grades differed from the non-teacher evaluations by family background and students’ school SES composition.

As hypothesised (hypothesis 1) and in line with previous research, our study indicates differences in the gender achievement gap in grades and standardised tests (Bonesrønning, 2008; Cornwell et al., 2013; Duckworth and Seligman, 2006; Falch and Naper, 2013; Lindahl, 2007; Protivínský and Münich, 2018; Matějů and Smith, 2015). We found that the gender achievement gap is smaller in non-teacher-evaluated PISA tests compared to teacher-evaluated grades in literacy. These estimated differences are similar to previous studies that have estimated gender grading bias to range from 0.2 to 0.5 SD in literacy and 0.1 to 0.3 in maths (Duckworth and Seligman, 2006; Lavy, 2008). However, in this study, we could not measure the “clean” effect of teacher bias because PISA tests and grades may not measure uniform aspects of learning, although previous studies have shown that students’ PISA test results and grades correlate very strongly in Finland (Pulkkinen and Rautopuro, 2022).

Our second hypothesis was that the gender achievement gap in grades would be the same as in the standardised test when we control for the effort put into school work and motivational factors. We assumed that girls (than boys) get higher (lower) grades compared to their PISA scores because girls (boys) have more (less) traits that are valued in schooling, such as being more interested in reading, more conscientious in doing homework, and putting more effort into schoolwork than boys. Indeed, our analyses support this hypothesis because measured factors explained about 0.27 SD of the literacy grade differences between girls and boys. The difference was even further reduced when we controlled for standardised test results (competence). However, even when the competence was controlled for in the models, only about 45% (0.34 SD) of the difference between genders could be explained. Even if we could explain away the so-called grading bias, there are still 55% unobserved differences between girls’ and boys’ literacy grades that we could not explain (0.43 SD). The results support the previous literacy that on average, boys’ schooling habits include putting less effort into schoolwork and reading compared to girls, and this is associated with boys’ grades, although their competence would be at the level of girls (Di Prete and Jennings, 2012; Heyder and Kessels, 2017). Thus, boys’ lower commitment to schoolwork explains teachers’ grading differences in test results. Teachers can also consider schoolwork in their grading, not only competence; on average, girls’ grades are higher than boys’. Furthermore, the remaining 55% unobserved difference in grading could be explained by boys’ classroom behaviour that we could not consider in this study. Previous literature has shown that boys’ behaviour is more disruptive than girls (Protivínský and Münich, 2018; Downey and Vogt Yuan, 2005; Terrier, 2020). In addition, boys have lower engagement in the classroom and interpersonal skills than girls, which could influence the results (Cornwell et al., 2013). Moreover, teachers tend to perceive girls on average as more motivated and better behaving than boys (e.g., Gentrup and Rjosk, 2018; Glock and Kleen, 2017).

Contrary to our hypothesis, the family background did not significantly reduce the gender achievement gap in grading or standardised test scores. However, as expected, the gender gap in literacy grades was lower only among high SES students than low SES students, but the differences between SES groups were marginal. The results show that girls received significantly better literacy grades regardless of their family background. We did not find strong support that family SES would be associated with boys’ counterculture in schools or that boy-typical culture influenced their grading or PISA test results.

However, schools’ SES composition of the students had a salient influence on the literacy grades of boys but not girls. We found teachers’ grading penalty for boys in schools with low SES students, although their effort, competence, and motivation would be at the same level as girls. Teachers’ perception of boys’ literacy skills in schools with many low SES students influences teachers’ grading of all boys.

This is in line with the previous studies, although previous research did not analyse how both teachers’ grading and standardised test scores depend on schools’ contexts (Legewie and Diprete, 2012; Machin and McNally, 2005). Furthermore, we did not find that schools’ students’ SES composition is associated with standardised test scores. Thus, we argue that boys’ masculine anti-school attitudes in lower SES schools cannot explain the gap between boys and girls. If boys’ anti-school attitudes and masculinity in low schools with low SES were to explain the results, we would also expect an effect on the standardised test scores. However, in lower SES schools, boys may receive lower grades than their competence and non-cognitive factors would predict because their habits do not align with the standards valued by the schools. We cannot exclude that boys’ behaviour can influence grading, particularly in schools where students’ SES composition is low, although many of the measured variables such as effort and competence can correlate with behaviour. Previous studies have found that particularly boys’ behaviour explains the achievement gap between girls and boys (Di Prete and Jennings, 2012; Trzesniewski et al., 2006). Future research should analyse how boys’ behaviour influences teachers’ grading in schools with low SES student composition.

This study has its limitations. One important thing is whether PISA scores and grades measure the same skills. The PISA tests measure the knowledge and skills that are relevant to the students’ future, whereas grades measure the competencies according to the primary school curriculum. Furthermore, part of our analyses was based on the PISA 2000 sample and students’ reporting. However, if there is no systematic bias between girls’ and boys’ reporting, our results can be considered robust. Our robustness analysis showed that this is the case because girls and boys got similar grades with similar PISA scores. Students reported average grades in the PISA 2000 sample did not differ from the registered school grades. In addition, stereotype threat could not explain the results.

Finally, using the *z*-score table, we calculated how much higher the boys’ grades would be if the competence was the same as in the PISA test results. The average math grades of the boys would be 8.2 instead of 7.5, and the average literacy grade would be 7.92 instead of 7.2. Boys’ grading penalty may influence later education choices and may result in boys being less likely to enter general secondary school than girls. The comprehensive school final grades strongly determine students’ educational paths in Finland. We consider this to be a significant problem from a gender-egalitarian perspective. Teachers should pay more attention to the grading practices of girls and boys in schools where students’ SES composition is low.

## Data Availability

The data analysed in this study is subject to the following licenses/restrictions: The Finnish register data is not available for the public. Requests to access these datasets should be directed to Statistics Finland.

## Footnotes

^1^The results conducted with students’ self-evaluated grades (PISA 2000 sample) did not deviate statistically significantly from the results conducted with grades from the school registers (see Appendix Figure 2A).
