# Supplementary material for: The gender achievement gap in grades and standardised tests—what accounts for gender inequality?
Source: Front Sociol. 2024 Oct 16;9:1448488. doi: 10.3389/fsoc.2024.1448488 (PMC11521978; doi:10.3389/fsoc.2024.1448488)
Supplement: Supplementary file 1 [file Data_Sheet_1.docx]

**APPENDICES**

**FIGURES**

**
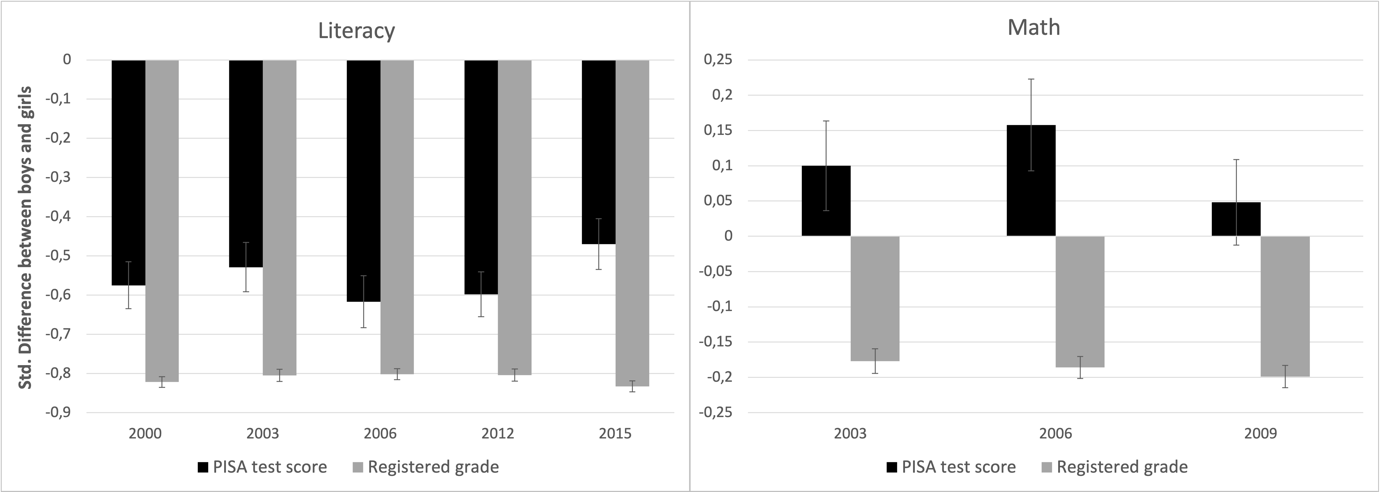
**

Figure 1A. Trends in the gender achievement gap in literacy in the years under study (2000-2015).


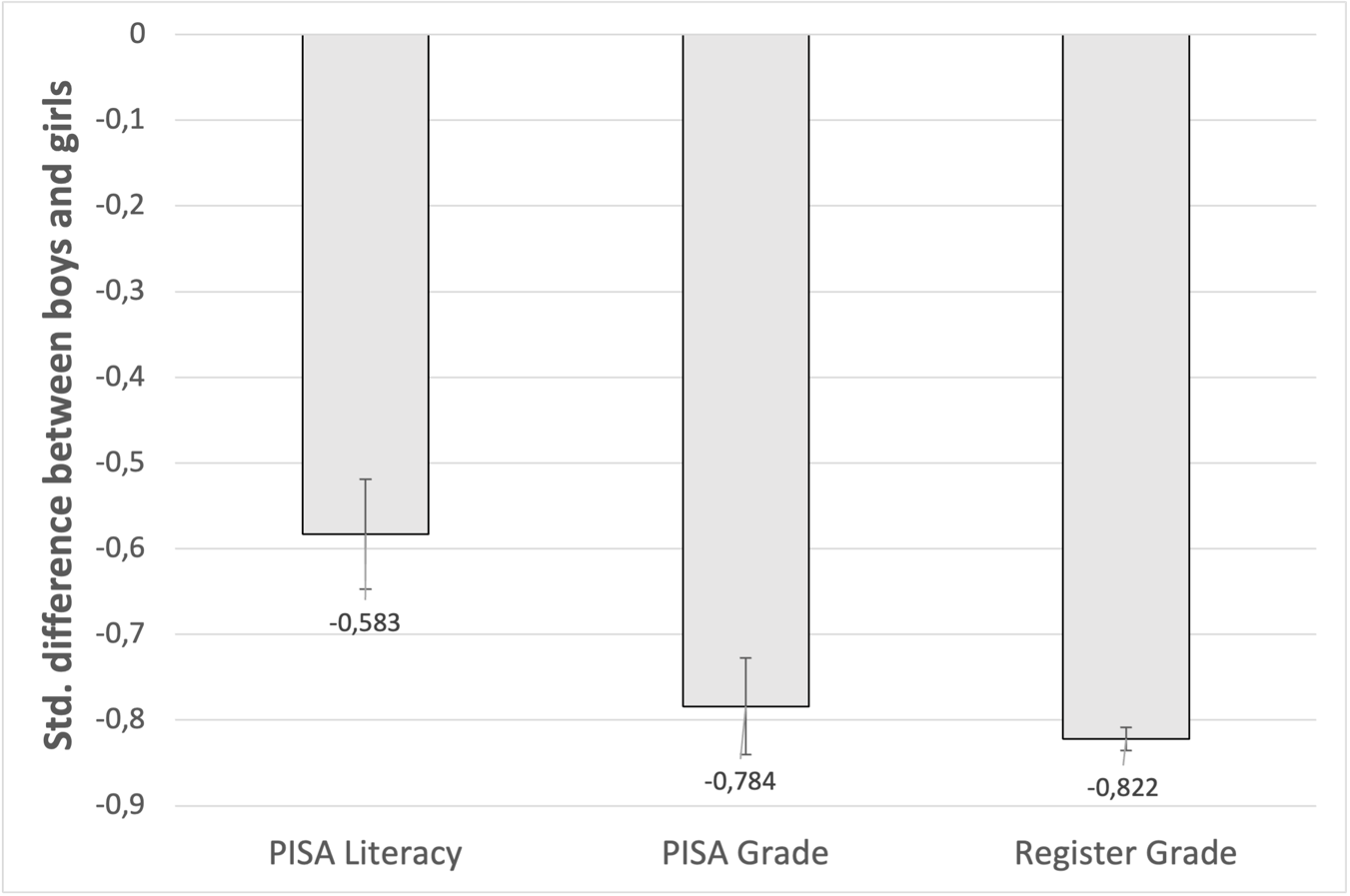


Figure 2A. The difference between boys’ and girls’ literacy PISA test scores, student-reported literacy grades in the PISA survey, and literacy grades from the school registers in 2000

Figure 3A. Differences between PISA test scores and grades in literacy of boys and girls by family background.


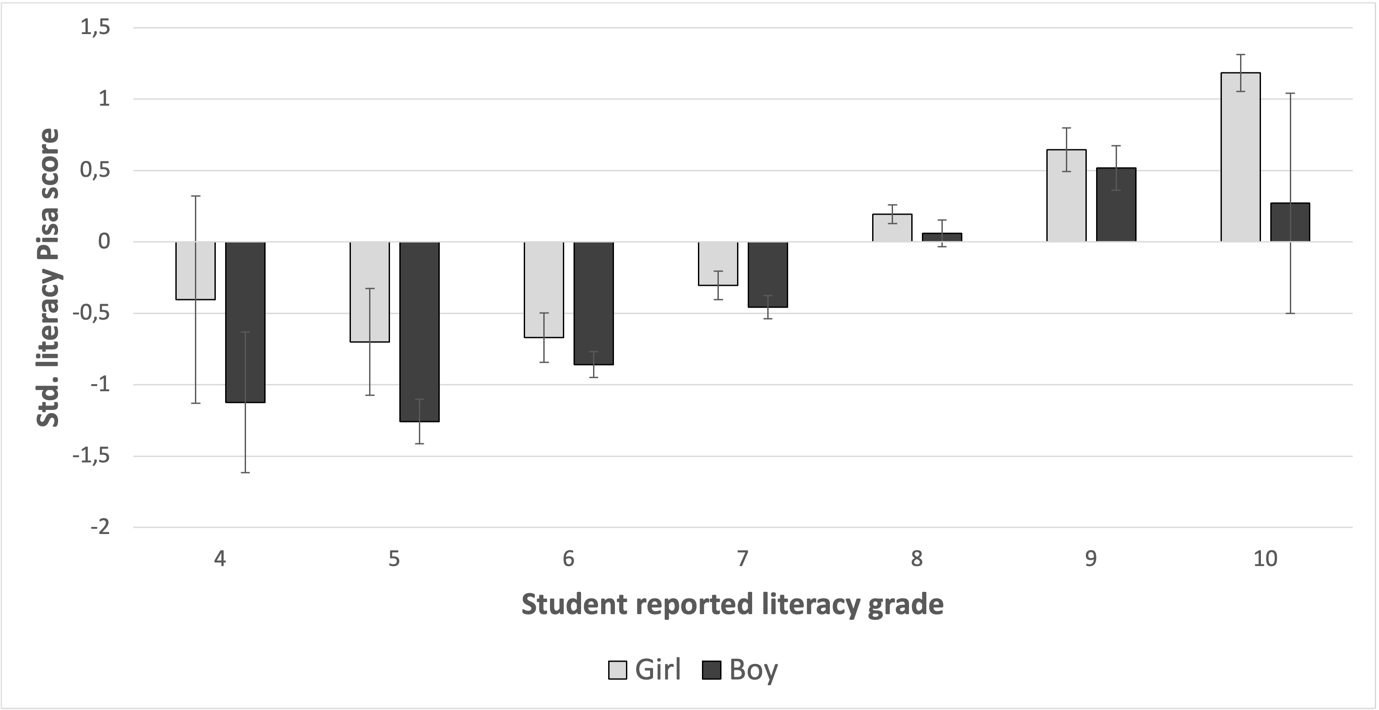


Figure 4A. Literacy score by student reported grade and gender in PISA 2000. Predicted score from OLS regression.

**TABLES**

Table 1A. Descriptive statistics

|  |  | Register | |  |  | PISA | |  |
| --- | --- | --- | --- | --- | --- | --- | --- | --- |
| Variable | Mean | Sd. | Min | Max | Mean | Sd. | Min | Max |
| Literacy (standardized) | 0,01 | 1,00 | -3,17 | 1,91 | 0,05 | 0,98 | -4,63 | 3,42 |
| Literacy | 7,74 | 1,20 | 4 | 10 | 540,97 | 87,23 | 109,63 | 840,78 |
| Parental ISEI | 49,18 | 16,86 | 16 | 90 | 51,50 | 18,55 | 12 | 90 |
| Parental ISEI centiles | 5,50 | 2,88 | 1 | 10 | 5,45 | 2,88 | 1 | 10 |
| Male | 0,51 | 0,50 | 0 | 1 | 0,50 | 0,50 | 0 | 1 |
| Immigrant | 0,02 | 0,14 | 0 | 1 | 0,03 | 0,16 | 0 | 1 |
| 2000 | 0,22 | 0,42 | 0 | 1 | 0,16 | 0,37 | 0 | 1 |
| 2003 | 0,20 | 0,40 | 0 | 1 | 0,19 | 0,40 | 0 | 1 |
| 2006 | 0,22 | 0,41 | 0 | 1 | 0,16 | 0,37 | 0 | 1 |
| 2012 | 0,16 | 0,37 | 0 | 1 | 0,29 | 0,45 | 0 | 1 |
| 2015 | 0,20 | 0,40 | 0 | 1 | 0,19 | 0,40 | 0 | 1 |
| *N* | 283677 | | | | 26702 | | | |

Table 2A. Test scores and grades for girls and boys in literacy and math

|  | Literacy | | Math | |
| --- | --- | --- | --- | --- |
|  | PISA  Test score | Grade | PISA  Test score | Grade |
| Girl | 0.325 | 0.422 | -0.0215 | 0.0988 |
| Boy | -0.235 | -0.39 | 0.0798 | -0.0853 |

Table 3A. Descriptive statistics for girls and boys of the PISA 2000 sample

| **GIRLS** | Mean | Sd. | Min | Max |
| --- | --- | --- | --- | --- |
| Literacy grade (std.) | 0,39 | 0,86 | -3,14 | 1,93 |
| Parental ISEI school mean | 13,66 | 6,64 | 0,00 | 34,31 |
| Parental ISEI | 33,91 | 16,32 | 0,00 | 74,00 |
| INTREA | 0,61 | 0,95 | -1,80 | 1,76 |
| EFFPER | 0,08 | 0,87 | -3,12 | 2,20 |
| Homework literacy | 1,81 | 0,78 | 0,00 | 3,00 |
| PISA score literacy (std.) | 0,28 | 0,93 | -4,43 | 3,23 |
| *N* | 2208 |  |  |  |
|  |  |  |  |  |
| **BOYS** | Mean | Sd. | Min | Max |
| Literacy grade (std.) | -0,39 | 0,97 | -3,14 | 1,93 |
| Parental ISEI school mean | 13,66 | 6,52 | 0,00 | 34,31 |
| Parental ISEI | 34,38 | 16,27 | 0,00 | 74,00 |
| INTREA | -0,24 | 0,93 | -1,80 | 1,76 |
| EFFPER | -0,13 | 0,89 | -3,12 | 2,20 |
| Homework literacy | 1,51 | 0,83 | 0,00 | 3,00 |
| PISA score literacy (std.) | -0,28 | 1,00 | -4,23 | 2,90 |
| *N* | 2008 |  |  |  |

Explanations of the variables of the table 3A:

**Parental ISEI school mean:** Mean of parental ISEI by schools

**Parental ISEI:** parental socioeconomic status measured in the ISEI scale

**EFFPER: Index of effort and perseverance:
(1 = almost never, 2 = sometimes, 3= often, 4= almost always)**

When studying, I work as hard as possible

When studying, I keep working even if the material is difficult

When studying, I try to do my best to acquire the knowledge and skills taught

When studying, I put forth my best effort.

**INTREA: Index of interest in reading**

**(1 = almost never, 2 = sometimes, 3= often, 4= almost always)**

Reading is fun

I wouldn’t want to give it up (reading)

I read in my spare time

When I read, I sometimes get totally absorbed

**HOMEWORK:** Complete literacy homework on time

Never 1
Sometimes 2

Most of the time 3

Always 4

PISA score literacy (std.): Z-standardized literacy PISA score

Table 4A. Linear regression interaction models between parental ISEI school mean and gender predicting literacy grade. Note uncontrolled model the below.

|  | Coefficient | SE | P-value |
| --- | --- | --- | --- |
| Boy | -0,53 | 0,066 | 0,00 |
| Parental ISEI school mean | 0,00 | 0,005 | 0,89 |
| Parental ISEI school mean x boy | 0,01 | 0,004 | 0,04 |
| Parental ISEI | 0,01 | 0,001 | 0,00 |
| INTREA | 0,08 | 0,020 | 0,00 |
| EFFPER | 0,21 | 0,019 | 0,00 |
| HOMEWORK | 0,17 | 0,019 | 0,00 |
| PISA score literacy (std) | 0,33 | 0,052 | 0,00 |
| Constant | -0,46 | 0,079 | 0,00 |
| *N* | 4216 |  |  |

Note: Model controls for immigration background

|  | Coefficient | SE | P-value |
| --- | --- | --- | --- |
| Boy | -0,95 | 0,063 | 0,00 |
| Parental ISEI school mean | 0,00 | 0,005 | 0,91 |
| Parental ISEI school mean x boy | 0,01 | 0,004 | 0,00 |
| Parental ISEI | 0,01 | 0,001 | 0,00 |
| Constant | -0,05 | 0,068 | 0,42 |
| *N* | 4216 |  |  |

Note: Models control for immigration background

Table 5A Linear regression interaction models between parental ISEI school mean and gender predicting standardized PISA literacy score. Note uncontrolled model the below

|  | Coefficient | SE | P-value |
| --- | --- | --- | --- |
| Boy | -0,26 | 0,08 | 0,00 |
| Parental ISEI school mean | 0,01 | 0,01 | 0,11 |
| Parental ISEI school mean x boy | 0,00 | 0,01 | 0,93 |
| Parental ISEI | 0,01 | 0,00 | 0,00 |
| INTREA | 0,30 | 0,02 | 0,00 |
| EFFPER | 0,12 | 0,02 | 0,00 |
| HOMEWORK | 0,05 | 0,02 | 0,01 |
| Constant | -0,56 | 0,12 | 0,00 |
| *N* | 4216 |  |  |

|  | Coefficient | SE | P-value |
| --- | --- | --- | --- |
| Boy | -0,65 | 0,08 | 0,00 |
| Parental ISEI school mean | 0,01 | 0,01 | 0,34 |
| Parental ISEI school mean x boy | 0,01 | 0,01 | 0,23 |
| Parental ISEI | 0,01 | 0,00 | 0,00 |
| Constant | -0,26 | 0,12 | 0,04 |
| *N* | 4216 |  |  |

Note: Models control for immigration background

Table 6A. Girls’ and boys' differences in literacy grade, PISA score, and the difference between girls' and boys' literacy grade and PISA score.

| **Parental ISEI mean within school** | **Girls’ and boys' difference in literacy grade** | **Girls' and boys’ differences in literacy PISA score** | **Difference between girls and boys difference** |
| --- | --- | --- | --- |
| **0** | 0,95 | 0,66 | 0,29 |
| **1** | 0,94 | 0,65 | 0,29 |
| **2** | 0,93 | 0,64 | 0,28 |
| **3** | 0,91 | 0,64 | 0,28 |
| **4** | 0,90 | 0,63 | 0,27 |
| **5** | 0,89 | 0,62 | 0,27 |
| **6** | 0,88 | 0,62 | 0,26 |
| **7** | 0,86 | 0,61 | 0,26 |
| **8** | 0,85 | 0,60 | 0,25 |
| **9** | 0,84 | 0,59 | 0,24 |
| **10** | 0,83 | 0,59 | 0,24 |
| **11** | 0,82 | 0,58 | 0,23 |
| **12** | 0,80 | 0,57 | 0,23 |
| **13** | 0,79 | 0,57 | 0,22 |
| **14** | 0,78 | 0,56 | 0,22 |
| **15** | 0,77 | 0,55 | 0,21 |
| **16** | 0,75 | 0,55 | 0,21 |
| **17** | 0,74 | 0,54 | 0,20 |
| **18** | 0,73 | 0,53 | 0,20 |
| **19** | 0,72 | 0,52 | 0,19 |
| **20** | 0,70 | 0,52 | 0,19 |
| **21** | 0,69 | 0,51 | 0,18 |
| **22** | 0,68 | 0,50 | 0,18 |
| **23** | 0,67 | 0,50 | 0,17 |
| **24** | 0,65 | 0,49 | 0,17 |
| **25** | 0,64 | 0,48 | 0,16 |
| **26** | 0,63 | 0,47 | 0,15 |
| **27** | 0,62 | 0,47 | 0,15 |
| **28** | 0,60 | 0,46 | 0,14 |
| **29** | 0,59 | 0,45 | 0,14 |
| **30** | 0,58 | 0,45 | 0,13 |
| **31** | 0,57 | 0,44 | 0,13 |
| **32** | 0,56 | 0,43 | 0,12 |

Table 7A. Correlations between the variables used in the study

|  | Literacy grade | Parental ISEI | Parental ISEI school mean | INTREA | EFFPER | HOMEWORK | PISA score literacy (std) |
| --- | --- | --- | --- | --- | --- | --- | --- |
| Literacy grade | 1,00 |  |  |  |  |  |  |
| Parental ISEI | 0,22 | 1,00 |  |  |  |  |  |
| Parental ISEI school mean | 0,13 | 0,41 | 1,00 |  |  |  |  |
| INTREA | 0,42 | 0,09 | 0,03 | 1,00 |  |  |  |
| EFFPER | 0,41 | 0,11 | 0,05 | 0,31 | 1,00 |  |  |
| HOMEWORK | 0,37 | 0,07 | -0,01 | 0,25 | 0,50 | 1,00 |  |
| PISA score literacy (std) | 0,53 | 0,24 | 0,15 | 0,42 | 0,26 | 0,21 | 1,00 |

***Appendix***

***The country context of the study***

In Finland, compulsory school begins at the age of 7 years and continues for 9 years. Education is free of charge and schools are funded through public finance. There are no private compulsory schools in Finland. PISA tests are conducted in the 9th grade when students get their final grades from compulsory school. We took the grades for literacy and mathematics from this year for this study. The comprehensive school leaving certificate grades are based on the teacher’s evaluation. Grades are evaluated by teachers in absolute terms thus teachers do consider the relative skill level of the class. PISA scores are based on standardized assessments that are not evaluated by teachers. Literacy grades were evaluated by the teachers in the final certificate of the compulsory school based on interpretation of the different texts, producing texts, understanding of the language, culture and literacy, and communicational skills Finnish National Agency For Education 2024). Grades for mathematics were evaluated based on mathematical skills including artihmetrics and geometrics skills and working skills (Finnish National Agency For Education 2024). The grading criteria sheet of the Finnish National Agency For Education does not mention explicitly that homework or class behavior would influence grading but we cannot exclude that teachers account for these kinds of activities in their grading also. Previous studies have shown that PISA literacy and mathematics are strongly correlated with grades evaluated by teachers. In this study correlation coefficients were between 0.5 – 0.6.

Finnish schools can be considered as female-dominated as approximately  80% of the teachers are women (Vipunen 2022). However, a previous study has shown the evaluation of students does not depend on the teacher’s gender in Finnish schools, although both male and female teachers evaluated grades for girls’ performance in the Finnish language as higher than boys’ performance (Krkovic et al. 2014). Also, similar findings have been reported by the most recent studies outside of Finland that find that teacher’s gender is not associated with the evaluation of the students and thus it has been argued that feminization of schooling is not causing the girl advantage in learning outcomes (Neugebauer, Helbig & Landmann 2011; Sokal, Katx, Chaszewski & Wojcik 2007; Driessen 2007; Holmlund & Sund 2008). The final grades from the compulsory school determine the students’ further educational paths because they are selected for academic or vocational secondary tracks based on the grades in the final certificate. As students’ future education is based on the final grades, it is important to investigate whether there is a gendered grading bias. Although we study the gender achievement gap in math and literacy here, we cannot take into account all academic grades that are evaluated for general secondary school, as the grades from these subjects are most important and correlate strongly with other academic subjects and educational attainment (Heiskala, Erola, and McMullin 2021).  The gendered grading bias can undermine boys entering general secondary school, which is an academic track for secondary education in Finland. For example, on average 57 to 58 percent of graduates from general secondary education (lukio) have been girls in recent years (2008–2020) (Statistics Finland 2022). Thus, girls overtake boys in the academic general secondary track.  More boys than girls temporarily discontinue school or drop out of secondary school altogether (Lehti, Erola, and Karhula 2019). One study showed that the transition phase to secondary education is the most crucial part of Finland's higher education and labor market attainment (Härkönen and Sirniö 2020). This is because in Finland students have to choose between general secondary and vocational educational paths of which the latter very rarely leads to university studies. According to the Global Gender Gap Report (World Economic Forum 2021), Finnish society is the second most gender-egalitarian in the world, and on average, women are better educated than men. Although Finland is famous for its quality schools, highly educated teachers, and high PISA results, school performance between girls and boys is highly segregated by gender, with the former having higher learning outcomes than the latter. The gender gap in PISA literacy and math tests is the highest among the OECD countries (OECD 2020; Sulkunen et al. 2010). The gender segregation of learning outcomes is demonstrated in Figure 1, which shows that the gender achievement gap in literacy and math scores is the greatest for girls in Finland among the OECD countries in PISA 2015.


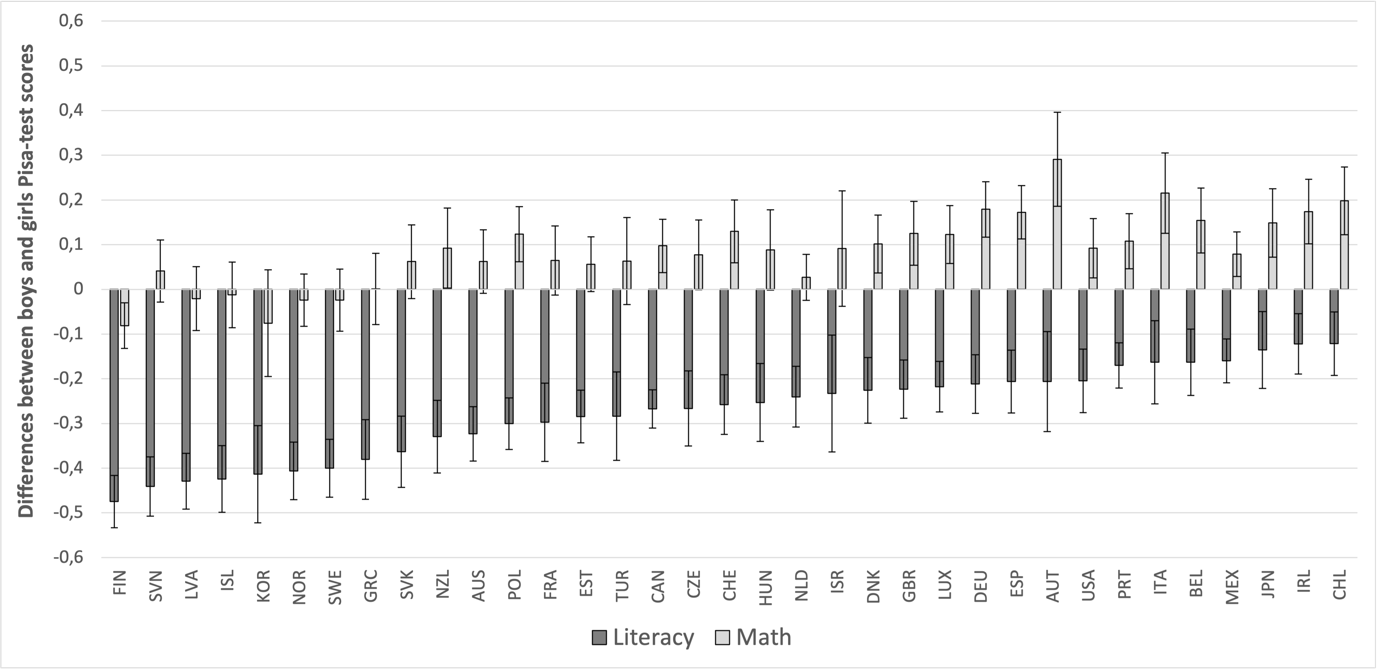


Figure 6A. The gender achievement gap in PISA literacy and math in OECD countries in 2015. Source: PISA 2015 data. Note Pisa-test scores are z-standardized and 95 % of CIs are around the estimates.

**References**

Driessen, G. (2007). The feminization of primary education: Effects of teachers’ sex on pupil achievement, attitudes and behavior. *International Review of Education*, *53*(2), 183-203.

Finnish National Agency For Education (2024): Äidinkielen ja kirjallisuuden päättöarvioinnin kriteerit. Retrevied form <https://www.oph.fi/fi/koulutus-ja-tutkinnot/aidinkielen-ja-kirjallisuuden-paattoarvioinnin-kriteerit>. 10.6.2024

Heiskala, Laura, Jani Erola, and Patricia McMullin. 2021. “Formal Differentiation at Upper Secondary Education in Finland: Subject-Level Choices and Stratified Pathways to Socio-economic Status and Unemployment.” *Longitudinal and Life Course Studies* 12(3):323–43.

Holmlund, H., & Sund, K. (2008). Is the gender gap in school performance affected by the sex of the teacher?. *Labour Economics*, *15*(1), 37-53.

Härkönen, Juho, and Outi Sirniö. 2020. “Educational Transitions and Educational Inequality: A Multiple Pathways Sequential Logit Model Analysis of Finnish Birth Cohorts 1960–1985.” *European Sociological Review* 36(5):700–19.

Lehti, Hannu, Jani Erola, and Aleksi Karhula. 2019. “The Heterogeneous Effects of Parental Unemployment on Siblings’ Educational Outcomes.” *Research in Social Stratification and Mobility* 64:100439.

Krkovic, Katarina, Samuel Greiff, Sirkku Kupiainen, Mari-Pauliina Vainikainen, and Jarkko Hautamäki. 2014. “Teacher Evaluation of Student Ability: What Roles Do Teacher Gender, Student Gender, and Their Interaction Play?.” *Educational Research* 56(2):244–57.

Neugebauer, M., Helbig, M., & Landmann, A. (2011). Unmasking the myth of the same-sex teacher advantage. *European sociological review*, *27*(5), 669-689.

Sokal, L., Katz, H., Chaszewski, L., & Wojcik, C. (2007). Good-bye, Mr. Chips: Male teacher shortages and boys’ reading achievement. *Sex roles*, *56*(9), 651-659.

Statistics Finland. 2022. “Completed Qualifications in Upper Secondary General Education, 2008–2020.” *Official Statistics of Finland.* Retrieved June 6, 2022 (<https://pxnet2.stat.fi/PXWeb/pxweb/en/StatFin/StatFin__kou__opiskt__lop/statfin_opiskt_pxt_135i.px/>).

Sulkunen, Sari, Jouni Välijärvi, Inga Arffman, Heidi Harju-Luukkainen, Pekka Kupari, Kari Nissinen, Eija Eija Puhakka, and Pasi Reinikainen. 2010. “PISA 2009 ensituloksia − 15-vuotiaiden nuorten lukutaito sekä matematiikan ja luonnontieteiden osaaminen*.* *Helsinki: Opetus- ja kulttuuriministeriön julkaisuja*” 2010:21.

Vipunen. 2022. “Personnel”*. Service Data of Finland and Finnish National Agency for Education*. Retrieved June 6, 2022 (<https://vipunen.fi/en-gb/basic/Pages/Henkil%C3%B6st%C3%B6.aspx>).

World Economic Forum. 2021. “Global Gender Gap Report 2021.” <https://www.weforum.org/reports/global-gender-gap-report-2021/>
